# Surfactant Effects on the Synthesis of Redox Bifunctional V_2_O_5_ Photocatalysts

**DOI:** 10.3390/ma13204665

**Published:** 2020-10-20

**Authors:** Islam Ibrahim, George V. Belessiotis, Michalis K. Arfanis, Chrysoula Athanasekou, Athanassios I. Philippopoulos, Christiana A. Mitsopoulou, George Em. Romanos, Polycarpos Falaras

**Affiliations:** 1 Institute of Nanoscience and Nanotechnology, National Centre for Scientific Research (NCSR) “Demokritos”, 15341 Athens, Greece; i.ibrahim@inn.demokritos.gr (I.I.); g.belessiotis@inn.demokritos.gr (G.V.B.); m.arfanis@inn.demokritos.gr (M.K.A.); c.athanasekou@inn.demokritos.gr (C.A.); g.romanos@inn.demokritos.gr (G.E.R.); 2 Department of Chemistry, Zografou, National and Kapodistrian University of Athens, 15784 Athens, Greece; atphilip@chem.uoa.gr (A.I.P.); cmitsop@chem.uoa.gr (C.A.M.)

**Keywords:** vanadium oxide, Tween 80 surfactant, photocatalysis, water pollutants, AOPs-ARPs

## Abstract

Novel V_2_O_5_ bifunctional photocatalysts were prepared following a wet chemical process with the addition of anionic or non-ionic surfactants into the precursor solution and further heating under reflux. Detailed characterization and investigation of the relevant light-matter interactions proved that surfactants addition had a strong impact on the morphology, while also affecting the crystallinity, the optoelectronic properties, and the surface chemistry of the novel photocatalysts. The most efficient photocatalyst (T80) was based on tween 80, a surface-active agent employed for the first time in the synthesis of vanadium oxide materials. T80 presented crystalline nature without structural defects, which are usually centers of e^−^ − h^+^ recombination. This material also exhibited small crystal size, high porosity, and short migration paths for the charge carriers, enabling their effective separation during photocatalysis. Under UV light illumination, T80 was capable to reduce hexavalent chromium to trivalent up to 70% and showed high yields in degrading methylene blue azo-dye and tetracycline antibiotic water pollutants. This remarkably high bifunctional performance defines T80 as a promising and capable photocatalytic material for both advanced oxidation and reduction processes (AOPs-ARPs).

## 1. Introduction

Industrial water always contains organic-inorganic pollutants of toxic and carcinogenic character. Because of their high solubility and stability, hexavalent chromium ions are among the major distinctively dangerous pollutants existing in wastewaters. In addition, artificial organic molecules (including dyes and antibiotics) that are generally used in our daily life and are discharged into water systems without actual treatment can cause water pollution [1,2,3,4]. As the need for clean water continues to be prominent around the world, there is increased interest in efficient photocatalysts. These materials can be activated using solar energy for the purpose of pollutant removal through oxidation or reduction [5]. TiO_2_ has been at the forefront of photocatalysis research since 1964 [6] however, there is increased interest in emerging novel materials. Aside from the route of synthesizing new materials, an equally promising direction is the investigation into the optimized synthesis of materials for the purpose of significantly enhancing their catalytic properties. A material previously thought to be unfit for photocatalytic applications can be modified to be an efficient future photocatalyst through the careful control and optimization of its characteristics during the preparation procedure.

One such promising material is V_2_O_5_. While it is most often used in applications such as batteries [7,8] or supercapacitors [9,10], it has shown promising photocatalytic behavior [11,12,13,14,15]. Many different morphologies of V_2_O_5_ have been studied both in the nanoscale (including nanobelts [16], nanowires [17] and nanorods [18]), but also in the microscale (such as microspheres [7,11]). Due to its layered structure, it is often intercalated with polyaniline [19] or lithium [20,21,22]. One of the most common V_2_O_5_ preparation methods (especially for photocatalytic applications) is the hydrothermal method [13,15,19]. Research into the effectiveness of V_2_O_5_ as a bifunctional photocatalyst, able to catalyze both oxidation and reduction reactions, has been relatively uncommon. In 2014, Roy et al. [12] synthesized V_2_O_5_ nanowires and used them for the effective oxidative degradation of dyes such as Methylene Blue. Raj et al. (2015) [13] prepared V_2_O_5_ nanoparticles hydrothermally with the use of surfactants SDS (Sodium Dodecyl Sulphate), CTAB (Cetyl Trimethyl Ammonium Bromide) and Triton-X. The samples prepared with SDS were used as photocatalysts for the Methyl Orange dye, achieving 84% degradation under UV light. Liu et al. (2015) [11] used hollow V_2_O_5_ microspheres for the degradation of gaseous 1, 2-dichlorobenzene (o-DCB) under visible light (degradation ratio 45% after 7 h). Aslam et al. (2015) [14] used V_2_O_5_ powder (synthesized with Triton X-100) under sunlight for the degradation of phenol and phenol derivatives. Lately, Jayaraj et al. (2018) [15] prepared V_2_O_5_ nanorods as photocatalysts under visible light for the treatment of Rhodamine 6G, Methyl Orange, and Methylene Blue, achieving 85%, 48%, and 24% degradation, respectively.

In general, even though several surfactants have been studied on the basis of their effects on the synthesis of vanadium pentoxide, there has been no comparison among surfactants in terms of nature and polarity, a common practice for TiO_2_ or SiO_2_ nanomaterials [23,24]. Specifically, studies on the effect of non-anionic surfactant Tween 80 on V_2_O_5_ are absent, despite the fact that its use improves the photocatalytic properties of TiO_2_ or ZnO [25,26]. For these reasons, we believe that the effect of surfactants during the synthesis of V_2_O_5_ is still a research area with plenty of potential. Thus, in this work, we employed different types of surfactants: sodium dodecyl sulphate (SDS), Tween 80, Triton X-100 (T100) and polyvinyl alcohol (PVA) for the synthesis of Vanadia-based photocatalysts. To our knowledge, the effect of promising non-ionic surfactant reagent T80-, which is known to improve the properties of photocatalysts such as TiO_2_ or ZnO on V_2_O_5_ nanomaterial synthesis, was examined for the first time. The primary target was to thoroughly investigate the effect of the surfactant’s nature on the morphological, structural, and opto-electronic properties of the prepared photocatalysts. The properties of the obtained materials were related to their photocatalytic efficiency for the degradation of model water pollutants, under both oxidation and reduction pathways. The materials prepared using Tween 80 present exceptional structural, morphological, and optoelectronic characteristics and showed the highest photocatalytic performance for the reduction of hexavalent chromium as well as the oxidation of methylene blue (azo-dye) and tetracycline (antibiotic) water contaminants.

## 2. Materials and Methods

### 2.1. Reagents and Materials

Ammonium vanadium oxide (NH_4_VO_3_, ≥99%) and potassium chloride (KCl, ≥99%) were obtained from Alfa Aesar (Athens, Greece), potassium hydroxide (KOH, ≥99%), polyvinyl alcohol (PVA-(C_2_H_4_O)x, ≥99%, molar mass = 86.09 g/mol) and diphenylcarbazide (DPC, 98%) were obtained from Merck (Athens, Greece), triton X-100 (T100 -C_14_H_22_O(C_2_H_4_O)_n_ (n = 9–10), molar mass = 647 g/mol), tween 80 (T80-C_64_H_124_O_26_, molar mass = 1.310 g/mol and potassium dichromate (K_2_Cr_2_O_7_, 98%) were purchased from Riedel-de Haen (Athens, Greece), while nitric acid (HNO_3_, 65%), absolute ethanol (C_2_H_5_OH) and hydrochloric acid (HCl, 37%) were supplied from Carlo Erba, VWR chemicals (Athens, Greece) and Fisher chemicals (Athens, Greece), respectively. Sodium dodecyl sulphate (SDS-NaC_12_H_25_SO_4_ molar mass = 288.37 g/mol), potassium bromate, potassium iodide, benzoquinone, and isopropyl alcohol, were purchased from Acros-Organics (Athens, Greece).

### 2.2. Materials Synthesis

The photocatalysts were synthesized under a convenient wet chemistry process as described in the literature [13], following a slight modification. Briefly, in a typical synthesis, 3.4 g of NH_4_VO_3_ were dissolved in a 1:1 ethanol:H_2_O mixture in the presence of appropriate quantities of PVA, SDS, T100 or T80 surfactants. The solution’s pH was first adjusted at ~2 using concentrated HNO_3_, and then it was maintained at 180 °C under stirring and reflux for 2 h. After cooling down, the catalysts were separated from the solution by filtration, and then consecutive washes with ethanol and H_2_O were followed. Lastly, the samples were calcinated at 400 °C for 2 h (ramp rate 5 °C/min), then raised to the temperature of 600 °C for another two hours in order to remove completely the surfactants from the synthesized materials. From now on, the samples will be labeled by surfactants’ name: PVA, SDS, T100, and T80.

### 2.3. Characterization Techniques

The structural properties of the samples were examined through X-ray diffractometry using a Siemens D500 diffractometer (Munich, Germany) (radiating at Cu K_a1_ λ = 1.5406 Å and Cu K_a2_ at 1.5444 Å). Additional crystallinity examination was performed with a Renishaw inVia Reflex micro-Raman spectrometer (New Mills, UK), equipped with a LEICA DMLM microscope (Wetzlar, Germany) and an emitting laser source operating at 785 nm. A Jeol JSM 7401F Field Emission Scanning Electron Microscopy (FE-SEM) (Tokyo, Japan), equipped with Gentle Beam mode, was used to observe the sample’s morphology. Moreover, the specific surface area (SSA) was determined with nitrogen adsorption-desorption isotherms at 77 K in an automated volumetric system (AUTOSORB-1-Krypton version-Quantachrome Instruments, Ashland, VA, USA)). The optical absorption was evaluated by the diffuse reflectance spectra of the samples, which was held with a UV-vis Hitachi 3010 spectrophotometer (Chiyoda-ku, Tokyo, Japan), equipped with an integrating sphere accessory and BaSO_4_ as reference. The surface properties of the synthesized materials were studied with IR spectroscopy using a Thermo Scientific Nicolet 6700 FTIR (Waltham, MA, USA) with an N_2_ purging system and the determination of zero charge point (PZC) under a batch equilibrium technique for each sample. In short, a specific amount of the samples was dispersed in six different KCl solutions (0.1 mol L^−1^) under vigorous stirring for 24 h, in which the pH medium was adjusted from acidic (using 0.1 mol L^−1^ HCl) to alkaline (using 0.1 mol L^−1^ KOH) [27]. In the end, the PZC was calculated by plotting the solution’s initial pH versus the final one.

### 2.4. Photocatalytic Experiments

In order to examine the effect of the surfactants on the activity of V_2_O_5_ photocatalysts, the degradation of three typical water pollutants was investigated: (i) the photocatalytic reduction of hexavalent chromium to trivalent (Cr(VI) − 5.8 × 10^−5^ mol L^−1^, pH solution adjusted to 2), (ii) the photocatalytic degradation of methylene blue (MB-3.1 × 10^−5^ mol L^−1^), and (iii) the photocatalytic oxidation of tetracycline (TC − 4.9 × 10^−5^ mol L^−1^). All the photocatalytic processes were performed into a black-box photoreactor, equipped with four UV-A Sylvania TLD 15W/08 lamps (Wilmington, MA, USA) (350–390 nm, 0.5 mW/cm^2^) and a cooling system [28]. In these experiments, the catalysts were first suspended into the pollutant’s solution under dark conditions until the sorption-desorption equilibrium was achieved. Then the photocatalytic experiments were carried out under UV-A irradiation. At constant time intervals, the catalyst was separated from the solution under centrifugation (Nahita Blue 261/1), and 4 mL of the supernatant solution was taken in order to determine the contaminant’s degradation kinetics with the UV-vis spectrophotometer. In all cases, 10 mg V_2_O_5_ were suspended into 10 mL of the respective pollutants, and the respective detection was accomplished at 542 nm for Cr(VI) (based on a typical colorimetric method, using the metal ion indicator DPC), 664 nm for the MB solution and 357 nm for the TC solution [29,30,31]. The photocatalytic degradation experiments were repeated in the presence of scavengers, in order to elucidate the mechanism and determine the reactive species in the photocatalytic processes. Specifically, potassium bromate (KBrO_3_), isopropyl alcohol (IPA), benzoquinone (BQ), and potassium iodide (KI) were used as electrons, hydroxyl radicals, anionic superoxide radicals, and hole quenchers, respectively.

## 3. Result and Discussion

### 3.1. Characterization

**Figure 1:**
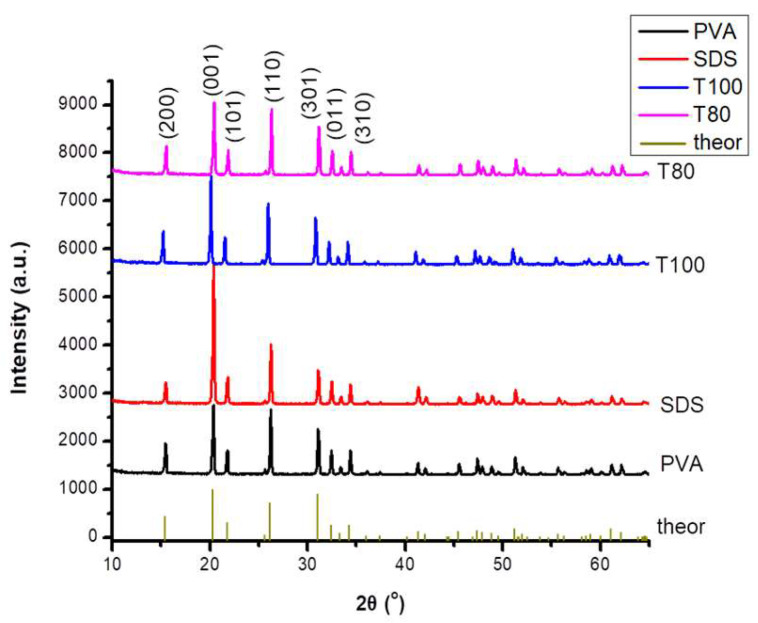
XRD diagrams for V_2_O_5_ samples prepared using PVA; SDS; T100 and T80 surfactants. The theoretical histogram of V_2_O_5_ is given as well.

X-ray powder diffraction patterns of the prepared V_2_O_5_ nanoparticles with different surfactants, noted as PVA, SDS, T100, and T80, generally present similar diffraction peaks, as shown in Figure 1. The main diffraction peaks, located at 15.4°, 20.3°, 21.7°, 25.6°, 26.2°, 31.0°, 32.5° and 34.3°, correspond to the (200), (001), (101), (201), (110), (400), (011) and (310), crystal planes, respectively. These diffraction peaks match well with the reported structure of the orthorhombic V_2_O_5_ (JCPDS Card no. 65–0131). No other vanadium oxide-based phases are observed, indicating that the V_2_O_5_ formed was of high purity. Considering the Scherrer’s equation for the most intense diffraction at 20.3°, it was estimated that the T80 sample has the lowest average crystallite size compared with the other samples, and particularly in the order T80 < PVA ≈ SDS < T100.

**Figure 2:**
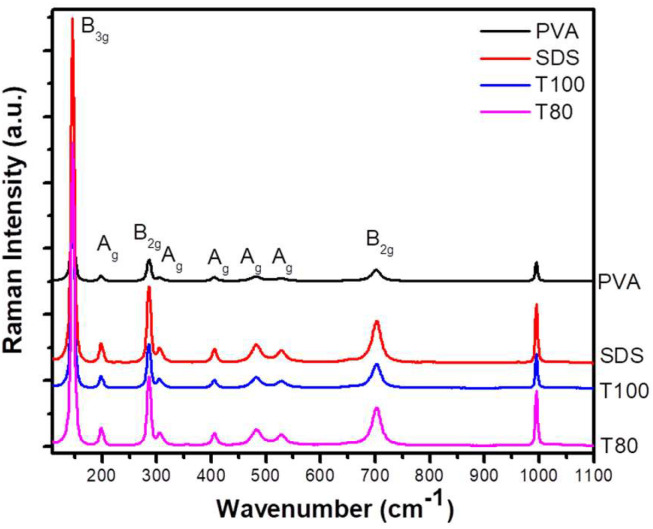
Raman spectra for V_2_O_5_ samples prepared using PVA; SDS; T100 and T80 surfactants.

For further insight into the structure of V_2_O_5_, Raman characterization was performed (Figure 2), verifying the samples high crystallinity and the absence of any impurities [17,18,20,21,32,33]. Nevertheless, the signal intensity was significantly lower for PVA, implying the sample’s relative lower crystallinity compared to the other materials. The most prominent band at 146 cm^−1^ is attributed to the skeleton bent vibration (B_3g_ mode), while the following modes at 198 cm^−1^ and 286 cm^−1^ can be connected to the bending vibrations of the O_C_–V–O_B_ bond (A_g_ mode and B_2g_ mode respectively). The bending vibration of triply coordinated oxygen V-O_C_ bonds (A_g_ mode) is responsible for the mode at 306 cm^−1^, while the A_g_ bending vibrations of V-O_B_-V bonds are detected at 405 cm^−1^ and at 482 cm^−1^. Next, the stretching modes of V–O_B_–V bonds (A_g_ mode) and V–O_C_ bonds (B_2g_ mode) are assigned at 529 cm^−1^ and 703 cm^−1^, respectively. Finally, the stretching mode of V = O double bonds leads to an intense peak at 995 cm^−1^ [17,18,20,21].

**Figure 3:**
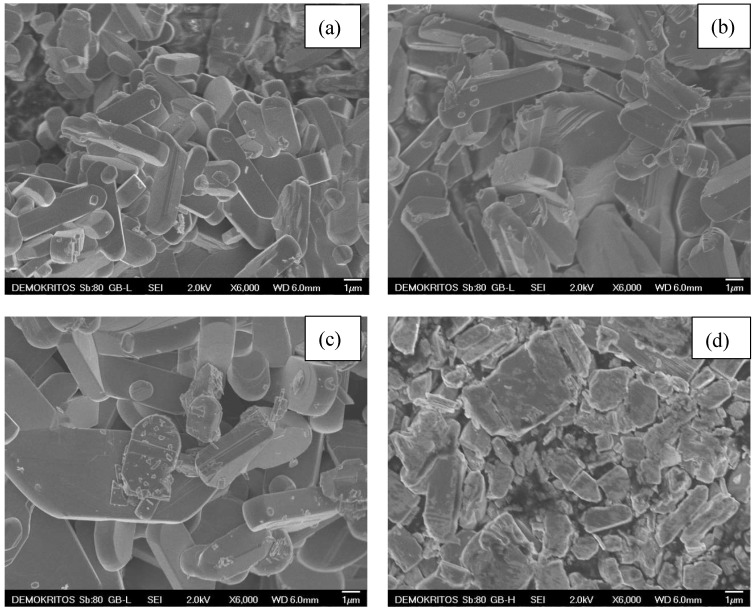
SEM images (in ×6000 magnification) of V_2_O_5_ samples prepared using Tween 80 (**a**); T100 (**b**); PVA (**c**); and SDS (**d**) surfactants.

The Scanning Electron Microscopy revealed significant differences among the samples, depending on the surfactant employed during the synthesis, as presented in Figure 3. First, tubular plates were formed for the T80 sample, with a smooth and homogenous surface (Figure 3a). Their length was altered with the studied crystal; nevertheless the dimensions were close to 1 × 1 μm^2^. By changing T80 with T100, another non-ionic surfactant with smaller molecular weight, the size of the plates was double-sized (Figure 3b).

A clear dependence between the non-ionic surfactants’ molecular weight and the morphology was evident when the even lighter PVA replaced the T80. These materials were much thicker, while some grains tended to lose that “tubular” morphology due to elongation in the 2-D (Figure 3c). As proof of these observations, the effect of the anionic surfactant SDS on the structure was also studied. In contrast with the other previous reagents, the polarity of this compound led to less homogenous structures, with random shapes and dimensions (Figure 3d).

**Figure 4:**
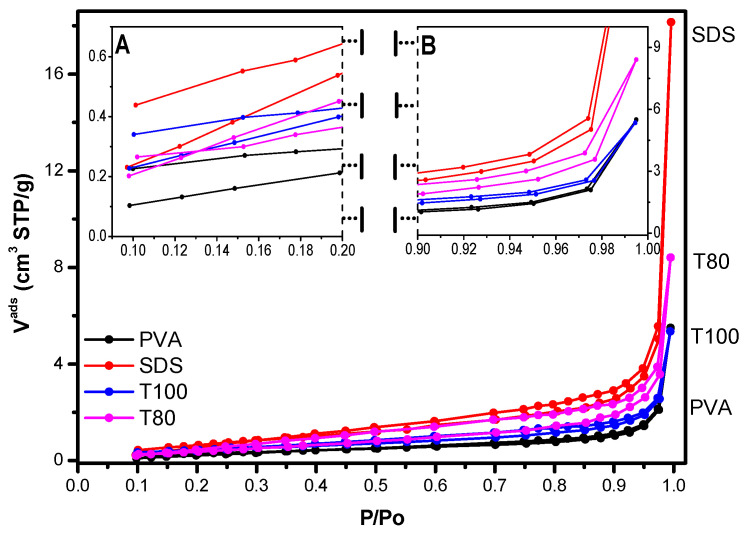
N_2_ porosimetry adsorption-desorption isotherms for PVA; SDS; T100 and T80 V_2_O_5_ samples.

N_2_ adsorption-desorption measurements at 77K were also conducted, and the obtained results are in good accordance with the morphological characteristics, as depicted by the SEM analysis. Table 1 presents the values of specific surface area (SSA) and total pore volume (TPV) for the synthesized V_2_O_5_ materials. All materials presented a reversible type II adsorption isotherm [34], characteristic of non-porous or macroporous materials (Figure 4). The shape of the isotherms, especially the lack of plateau at high relative pressures, indicates the absence of a well-defined mesopore volume and confirms the dominance of macropores (>50 nm), which represent the empty space nesting between the aggregates of V_2_O_5_ crystals. The T80 sample and the sample prepared using the anionic surfactant (SDS) especially exhibited a larger BET surface area and higher adsorbed amount of N_2_ at P/P_0_ = 0.995 as compared to the samples derived with the T100 and PVA surfactants. These properties are indicative of the smaller crystal size in samples T80 and SDS and confirm the output of SEM analysis (Figure 3), though at a macroscopic scale which is representative of the entire sample. Hence, since for all practical purposes the upper limit of reliable measurement using N_2_ adsorption at 77K is around a P/P_0_ value of 0.995, which corresponds to a pore size of around 400 nm, the higher amount of N_2_ adsorbed on samples T80 and SDS at P/P_0_ = 0.995 indicates that these two samples possess a larger fraction of pores of size in the area of 400 nm (where capillary condensation can take place).

Considering the output of SEM analysis (crystal dimensions close to 1 × 1 μm^2^), this comes in convergence with the rule of 2.5:1 (particle size:interparticle space size) which holds in general for the mean dimension of the interparticle voids existing in aggregates of particles. It is also interesting that while the crystals of the SDS and T80 samples have almost identical mean dimensions (Figure 3a,d), SDS exhibits wider crystal size distribution and possesses crystals of rougher surface texture. The latter property is reflected by the higher BET surface area of SDS as compared to T80, while the wider size distribution leads to a denser packing of crystals, and therefore, to a larger volume of macropores with appropriate size for capillary condensation to take place. Therefore, the adsorbed amount of N_2_ on SDS is higher than that on T80. Finally, it can be seen (Figure 4) that amongst the prepared samples, only T80 and SDS presented type H3 hysteresis loops [30], which are associated with aggregates of platy particles, (crystals in our case). As indicated in Figure 4, although only the initial monolayer–multilayer section of the isotherms is reversible, the whole adsorption branch of the H3 loop appears to exhibit the same shape as a type II isotherm. This pseudo-type II character is associated with the metastability of the adsorbed multilayer (and delayed capillary condensation) and is due to the low degree of pore curvature and non-rigidity of the aggregate structure.

**Figure 5:**
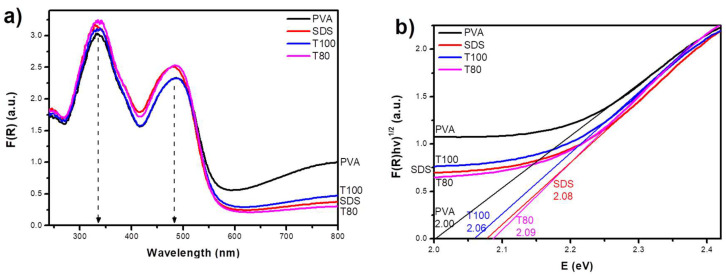
Optical absorption spectra for PVA, SDS, T100, and T80 V_2_O_5_ samples (**a**); respective Tauc plots and estimated band gaps of the synthesized materials (**b**).

The prepared samples presented a typical vanadium pentoxide optical absorption spectrum (Figure 5a), extending almost to 600 nm, in good agreement with the literature [14,15,35].

The profound strong absorption in the UV and the visible region is representative of the charge transfer from the valence band, comprised of O_2p_ orbitals, to the empty V_3d_ bands of the conduction band. Moreover, an additional source of enhanced absorption in the visible region might arise from the electronic transitions between the splitted d orbitals (*t*_2g_ to *e*_g_)” [14,31]. The exact origin of absorption maxima at ~335 and ~480 nm is not clearly defined; nevertheless, the different ion coordination in the crystal was previously proposed.

**Figure 6:**
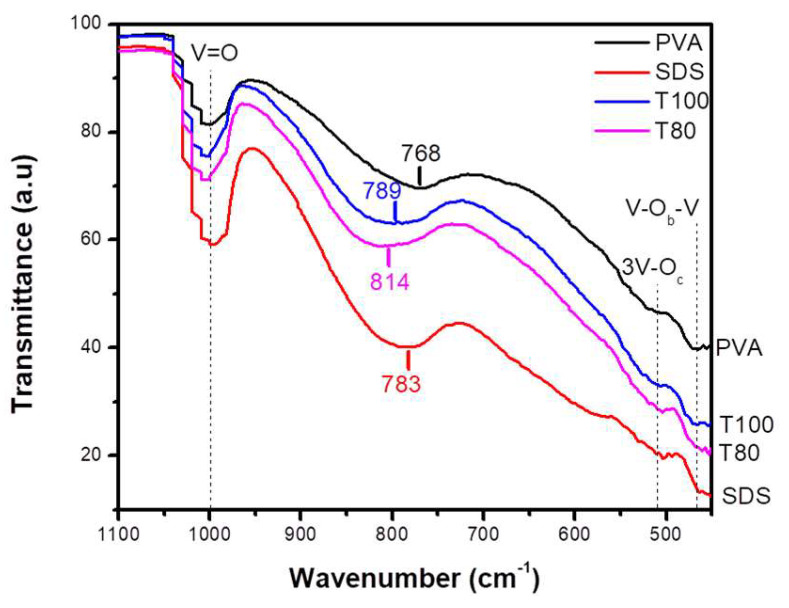
FTIR spectra for V_2_O_5_ samples with PVA; SDS; T100; T80 surfactants.

The V_2_O_5_ investigation through FT-IR spectroscopy was mainly focused between 400 and 1050 cm^−1^, as various vibrations of “V–O” type groups can be identified in this wavelength range. The samples’ spectra in Figure 6 present four significant peaks, all of which are in satisfactory agreement with the literature data [16,19,36,37] as a possible explanation [15]. Considering the indirect bandgap type of V_2_O_5_ photocatalysts, they were estimated to be equal to 2.0 eV for PVA, 2.14 eV for SDS, 2.12 eV for T100, and 2.05 eV for T80, using the Tauc plots in Figure 5b.

Specifically, the leftmost and most intense peak at ~1000 cm^−1^ can be attributed to the stretching vibration of the V = O bond, and possibly the asymmetric stretching vibration of the V–O_b_–V (O_b_, bridge oxygen) group leads to the broad modes in the area of 768−814 cm^−1^ [16,32]. Finally, the smaller vibrational peaks at the range 503 to 519 cm^−1^ and at ~465 cm^−1^ are present, possibly due to 3V–O_c_ stretching (O_c_: threefold coordinated oxygen atom) and V–O_b_–V bridging deformations [16,32,33]. It is pointed out that no signal corresponding to organic compounds or carbonic complexes was detected above 1100 cm^−1^, implying that the organic precursors were totally removed during the annealing and the catalysts’ surfaces were free from any organic residuals.

**Figure 7:**
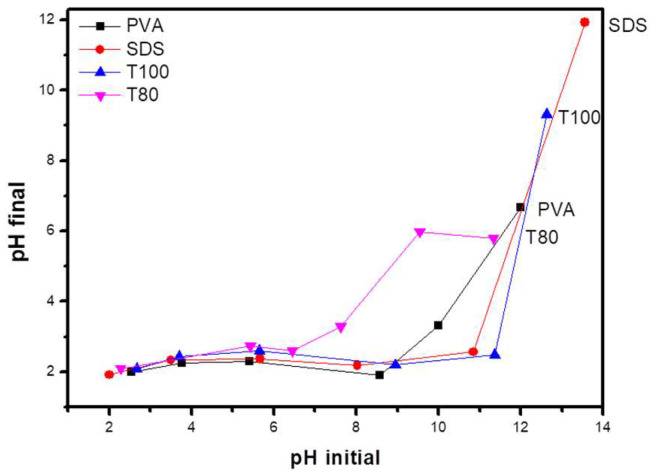
Determination of PZC for the synthesized V_2_O_5_ samples.

Moreover, the determination of the zero charge point (PZC) for the synthesized catalysts improved the understating of their surface chemistry. By plotting the initial versus the final pH values (Figure 7), the exact PZC values fluctuated from 1.9 to 2.1. In any case, these values are in agreement with the literature [38], and, therefore, the samples’ surface will be charged positively when the samples are immersed into solutions with pH less than 2 (such as the used Cr(VI) solution) or negatively for solutions with higher pH (e.g., for the used MB and TC solutions).

### 3.2. Photocatalytic Activity and Trapping Experiments

In order to evaluate the photocatalytic disinfection properties of the new V_2_O_5_ catalysts, it was important to examine both reduction and oxidation photocatalytic reaction pathways. For this reason, Cr(VI), an industrial and carcinogenic pollutant, was used for the reduction processes. Likewise, methylene blue, a very common azo-dye in textiles, and tetracycline, a recalcitrant antibiotic contaminant in water treatment, were selected as target pollutants for the oxidation processes.

#### 3.2.1. Advanced Reduction Processes (ARPs)

**Figure 8:**
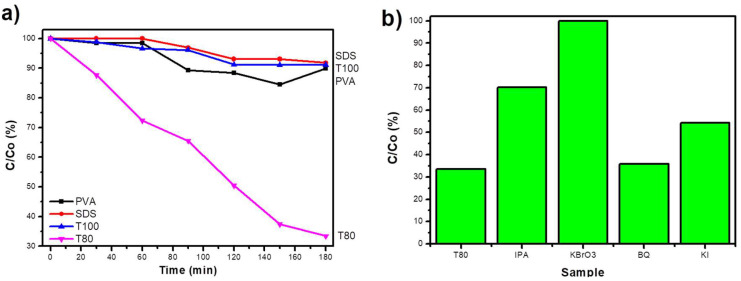
The photocatalytic reduction activity of Cr(VI) using the synthesized V_2_O_5_ samples (**a**); the effect of scavengers on the photocatalytic reduction of Cr(VI) by T80 (**b**).

The photocatalytic performance was first evaluated with hexavalent chromium, as shown in Figure 8a. Indicatively, the solution’s pH was equal to 1.7, so the catalyst’s surface was positively charged and the chromium complexes adsorption should be promoted. Moreover, the conduction band potential of V_2_O_5_ is less negative than the chromium redox potential from hexavalent to trivalent [39], thus, the reduction should occur as follows: Cr_2_O_7_^2−^ +6e^−^ + 14H^+^ → 2Cr^3+^ + 7H_2_O, E^0^_Cr(VI)/Cr(III)_ = 1.33 V.

However, the observed absence of photocatalytic activity for PVA, SDS, or T100 was not abnormal, since V_2_O_5_ materials are usually photocatalytically inert or they can partially remove Cr(VI) [40,41]. Therefore, the efficient photocatalytic reduction of Cr(VI) to Cr(III) up to 70% after 180 min with T80 was astonishing. As long as all the samples have similar structural properties and surface chemistry, the superior behavior of T80 might be related to its morphology. It is assumed that the smaller grain size favors better charge carriers’ separation, so there are more reactive species available for the reduction of chromium ions. In order to verify which reactive species were responsible for the photocatalytic reduction of Cr(VI), the experiments with T80 were repeated in the presence of scavengers. As presented in Figure 8b, the photocatalytic efficiency was totally hindered when KBrO_3_ was added, whereas the addition of the other scavengers did not affect it significantly, demonstrating that the photogenerated electrons (e^−^) are the main active species and responsible for the Cr(VI) reduction.

#### 3.2.2. Advanced Oxidation Processes (AOPs)

**Figure 9:**
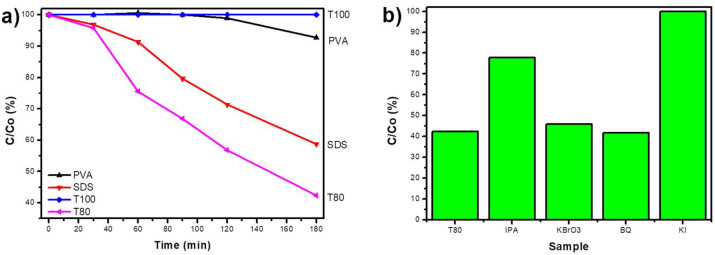
The photocatalytic degradation of MB with the synthesized materials (**a**); the photocatalytic degradation of MB with the most efficient photocatalyst, T80, in the presence of appropriate scavengers (**b**).

MB and TC were selected as appropriate organic target pollutants with the intention of testing the capability of V_2_O_5_ photocatalysts to degrade them through the oxidation path. Figure 9a shows that only SDS and T80 were able to provoke the MB solution discoloration during UV-A irradiation (any adsorption effects were already eliminated during the sorption-desorption equilibrium).

T80 was the only photocatalyst synthesized with non-anionic surfactant, which presented an MB concentration decrease up to 40% after 3 h. On the other hand, even if SDS was a functional catalyst, it was less efficient than T80. Finally, the scavengers’ experiments proved that the main reactive species were the photogenerated holes (h^+^ = KI quencher); however, the hydroxyl radicals (·OH = IPA quencher) had also a significant role in the photocatalytic process (Figure 9b).

**Figure 10:**
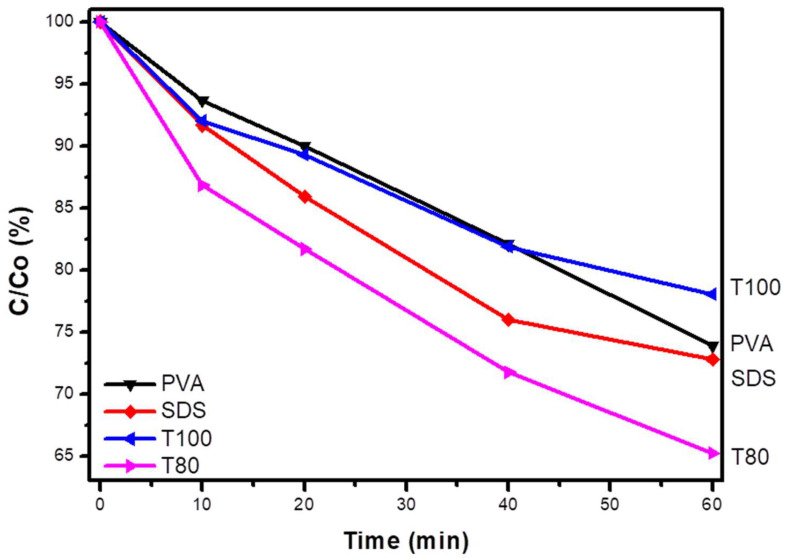
The photocatalytic degradation of TC using the synthesized materials.

Furthermore, tetracycline (TC) was selected as a representative water pollutant to evaluate the oxidation activity of the new V_2_O_5_ photocatalysts against antibiotics. The photocatalytic degradation kinetics of TC are reported in Figure 10. The reaction mechanism is based on the compound’s oxidation by holes and hydroxyl radicals [27], and the obtained results confirm that the T80-based materials present the best removal yields, almost 10% higher than the samples prepared with the other surfactants. This offers additional evidence for the high ability of T80 performance in photocatalytic oxidation processes against contaminants in water.

Tween 80 is a hydrophilic nonionic surfactant which possesses high solubilizing ability. It helps to dissolve and stabilize the ethanol:water reaction mixture containing the NH_4_VO_3_ precursor. Solubility experiments were performed which confirmed the higher ability of the T80 surfactant to solubilize the etanol:water mixture containing the NH_4_VO_3_ precursor. This is probably due to the fact that T80 disposes of the sorbitan assembly with a high number of hydrophilic polyoxyethylene groups. Indeed, the maximum solubility value of NH_4_VO_3_ in the presence of T80 was 0.21 mol L^−1^, about 3 times higher than the corresponding values for saturated solutions in the presence of T100, SDS and PVA surfactants, respectively. The obtained photographs (not shown) clearly indicate the absence of any precipitate, especially for the 0.21 mol L^−1^ solution containing T80. On the contrary, this is not the case of equimolar solutions without surfactant and those containing T100, SDS, and PVA, where a significant amount of sediment exists. As T80 is also a well-known emulsifier, its presence helps the reaction ingredients mix together and prevent the separation of the particles. As a result, its use leads to better control of the morphology of the resulting T80 vanadium pentoxide photocatalysts, a fact that was confirmed by the corresponding SEM analysis. Such improved morphology and crystallinity together with high surface area and pore volume can be at the origin of the enhanced photocatalytic performance observed for the T80 materials.

## 4. Conclusions

Novel V_2_O_5_ photocatalysts were prepared under convenient wet chemistry routes using a reflux system and a simple calcination step. Four different materials were synthesized by adding different surfactants (PVA, SDS, T100, and T80) into the precursor solution. All samples exhibited similar crystallinity, optoelectronic, and surface chemistry properties, while the T80 sample presented the smaller crystal grains with the higher porosity, suggesting an inverse proportional correlation between morphology and the non-ionic surfactant’s molecular weight. Moreover, the T80 sample exhibited a remarkable photocatalytic performance against a wide range of pollutants, such as Cr(VI), MB, and TC, and therefore is suggested as a very efficient catalyst for both Advanced Reduction and Oxidation Processes.

## Figures and Tables

**Table 1:** Estimated specific surface area (SSA) and total pore volume (TPV) for the synthesized V_2_O_5_ materials.

Surfactants (Samples Name)	SSA (m²/g)	TPV (mL/g)
PVA	1.18	3.25 × 10^−3^
SDS	3.13	7.77 × 10^−3^
T100	1.73	3.95 × 10^−3^
T80	2.10	5.25 × 10^−3^
